# Fatal Effects from Carelessness of an Apothecary

**Published:** 1850-08

**Authors:** 


*Fatal effects from Carelessness of an Apothecary.*—A Boston (Mass.) Apothecary lately put up ten grs. of the *oxy*-muriate of mercury for the *sub*-muriate, which was written for by a physician. It was taken by the patient, and proved fatal. The Suffolk District Medical Society, in view of this occurrence, have appointed a committee to consider, and report upon the expediency of dispensing with the use of Latin in writing medical prescriptions. The mistake, however, in this instance, could not have consisted in reading the prescription wrongly, as no apothecary of decent information would suppose that ten grains of the oxy-muriate would be prescribed for a patient. The carelessness must have been in giving the wrong article, and would probably have happened if the term *calomel* had been used.

